# Shedding light on carboxyhaemoglobin in extracorporeal membrane oxygenation

**DOI:** 10.1186/s13054-023-04671-8

**Published:** 2023-10-03

**Authors:** Kenneth R. Hoffman, Arne Diehl, Eldho Paul, Warwick Butt, Aidan J. C. Burrell

**Affiliations:** 1 Intensive Care Unit, Alfred Hospital, Melbourne, Australia; 2 School of Public Health and Preventive Medicine, Monash University, Melbourne, Australia; 3 Australian and New Zealand Intensive Care Research Centre, School of Public Health and Preventive Medicine, Monash University, Melbourne, Australia; 4 Intensive Care Unit, The Royal Children’s Hospital, Melbourne, Australia; 5 Department of Critical Care, Faculty of Medicine Dentistry and Health Sciences, University of Melbourne, Melbourne, Australia; 6 Paediatric Intensive Care, Murdoch Children’s Research Institute, Melbourne, Australia

Dear Editor,

Carboxyhaemoglobin has been proposed as a novel marker of haemolysis in extracorporeal membrane oxygenation (ECMO). It is produced by the endogenous production of carbon monoxide from the breakdown of haem by the enzyme haem oxygenase. In a previous pilot study, we demonstrated a close correlation between carboxyhaemoglobin and plasma free haemoglobin, the gold standard for diagnosing intravascular haemolysis, in four patients with acute severe haemolysis on ECMO [1]. However, questions remain about the validity of this relationship.

To investigate this further, we collected 880 simultaneously sampled measurements of carboxyhaemoglobin and plasma free haemoglobin in 29 adult patients supported on veno-venous ECMO. These samples were collected in the 72 h before and after a circuit and membrane oxygenator exchange for circuit dysfunction, which occurs commonly with circuit haemolysis. Carboxyhaemoglobin increased from a baseline of 1.4% to a peak of 1.8% (*p* < 0.01), representing a relatively large increase in blood carbon monoxide content. However, this attenuated increase in carboxyhaemoglobin did not match the 42-fold increase in plasma free haemoglobin (0.012–0.501 g/L *p* < 0.01). The weighted correlation coefficient was low at 0.29 and did not reach statistical significance (*p* = 0.13).

Interference with co-oximetry from bilirubin, haemolysis and lipaemia [2], or the presence of unmeasured dissolved carbon monoxide may be proposed as contributors to the lack of increase in carboxyhaemoglobin we observed. However, we hypothesise that exposure of blood in the ECMO circuit to visible light may be causing the carbon monoxide to dissociate from haemoglobin [3, 4]. This may contribute to a reduction in the half-life of carboxyhaemoglobin, particularly when combined with high oxygen partial pressure within the ECMO circuit and clearance through both pulmonary ventilation and extracorporeal gas exchange.

Light on the visible spectrum is known to dissociate carbon monoxide from haemoglobin, an effect first described by Haldane and Smith in 1896 [3]. The clinical utility of this effect has not been studied extensively, although there is emerging animal data suggesting that exposure to visible light via an extracorporeal circuit may be used to treat carbon monoxide poisoning [4].

To date however, there is a lack of human data showing the potential impact that visible light may have on chemical reactions in the blood during ECMO. This phenomenon could have widespread clinical implications for patients supported with ECMO. In addition to carboxyhaemoglobin, photodegradation of parenteral drugs is well described and includes essential classes of antibiotic, antifungal and immunosuppressant medications such as cephazolin, amphotericin and hydrocortisone. Studies that describe unusual pharmacokinetics during ECMO may make assumptions that there is binding of the drug to the circuit and oxygenator. However, photodegradation may also contribute. Additionally, exposure to light may have haematological effects on red blood cell membrane integrity and platelet function [5].

Understanding the physiological impact of ECMO on the body is a high priority for clinicians delivering ECMO. Further research is needed to understand the effects that light may have on the extracorporeal blood, including chemical reactions, drug potentiation and inactivation.

## Data Availability

Original data are available on request.
